# 1990. Evaluating the Impact of Directed Provider Outreach Urging Polio Vaccination During a Polio Outbreak – Rockland County New York, 2022

**DOI:** 10.1093/ofid/ofad500.117

**Published:** 2023-11-27

**Authors:** Nina B Masters, Hannah Fast, Sarah Hamid, James Lueken, Shani K Doss, Adina L de Coteau, Dina Hoefer, Haillie Meek, Neida Mita-Mendoza, Susan Miller, Kevin T McKay, Chitra Punjabi, Maria Souto, Tatiana De Luna-Evans, Shannon K Stokley, Kathleen L Dooling

**Affiliations:** Epidemic Intelligence Service, Centers for Disease Control and Prevention; Division of Viral Diseases, National Center for Immunization and Respiratory Disease, CDC, Atlanta, GA; Immunization Services Division, National Center for Immunization and Respiratory Disease, CDC, Atlanta, Georgia; Epidemic Intelligence Service, Centers for Disease Control and Prevention; Coronavirus and other Respiratory Viruses Division, National Center for Immunization and Respiratory Disease, CDC, Atlanta, Georgia; Immunization Services Division, National Center for Immunization and Respiratory Disease, CDC, Atlanta, Georgia; Immunization Services Division, National Center for Immunization and Respiratory Disease, CDC, Atlanta, Georgia; Immunization Services Division, National Center for Immunization and Respiratory Disease, CDC, Atlanta, Georgia; New York State Department of Health, Albany, NY; New York State Department of Health; Epidemic Intelligence Service, Centers for Disease Control and Prevention, Albany, New York; New York State Department of Health, Albany, NY; New York State Department of Health, Albany, NY; Rockland County Department of Health, Pomona, NY; Rockland County Dept of Health, Pomona, New York; Rockland County Department of Health, Pomona, NY; Rockland County Dept of Health, Pomona, New York; Immunization Services Division, National Center for Immunization and Respiratory Disease, CDC, Atlanta, Georgia; Centers for Disease Control and Prevention, Atlanta, Georgia

## Abstract

**Background:**

On July 18, 2022, a case of paralytic polio was reported in a person from Rockland County, New York. In August 2022, the proportion of children in Rockland County with 3 doses of polio vaccine by age two was 60.3%. That month, Rockland County, New York State, and CDC initiated a campaign to conduct physician-mediated patient outreach to improve Inactivated Polio Vaccine (IPV) coverage in Rockland County.

**Methods:**

We contacted 38 Vaccines for Children enrolled providers, serving patients aged 3-60 months who were overdue for polio vaccinations, to send reminders to parents. Providers contacted parents by phone, text message, or mailed personalized letter. To assess success of the intervention on vaccine uptake, we retrospectively identified cohorts of patients missing >1 dose of polio vaccine in the New York State Immunization Information System as of August 9, for baseline years 2017 and 2018, and for intervention year 2022. We used logistic regression to compare the odds of receiving polio vaccine among under-vaccinated cohorts during August 15–October 31 of the baseline and intervention years.

**Results:**

Thirty providers conducted patient outreach using phone calls (19 providers), text messages (4 providers), and letters (14 providers). At the outset, 4,887 patients aged 3-60 months were missing 1 or more recommended doses of polio vaccine. During the 10 weeks following initiation of outreach, 1,292 (26.4%) of these patients received an IPV dose; 7.1% were 3-6 months old, 17.9% 7-11 months, 27.9% 1 year, 47.1% 2-5 years. Of the IPV doses received, 1^st^, 2^nd^, and 3^rd^ doses accounted for 7.5%, 33.1% and 59.4%, respectively. The odds of an un- or under-vaccinated patient receiving a dose of polio vaccine during the 10-week analysis period were significantly higher in 2022 compared to 2017 (OR=1.41, 95% CI [1.27-1.56]) and 2018 (OR=1.46, 95% CI [1.32-1.62]).

**Conclusion:**

Following physician-mediated patient outreach urging vaccination among under-immunized children in August 2022, 26% of undervaccinated children received polio vaccine in the 10 weeks after outreach. This reflected an increased odds of vaccination by 40% compared to baseline years 2017 and 2018, highlighting the positive impact of conducting this type of outreach during a polio outbreak in an area with low vaccine coverage.

**Disclosures:**

**All Authors**: No reported disclosures

